# Interleukin-33 and thymic stromal lymphopoietin, but not interleukin-25, are crucial for development of airway eosinophilia induced by chitin

**DOI:** 10.1038/s41598-021-85277-4

**Published:** 2021-03-15

**Authors:** Ken Arae, Masashi Ikutani, Kotaro Horiguchi, Sachiko Yamaguchi, Youji Okada, Hiroki Sugiyama, Keisuke Orimo, Hideaki Morita, Hajime Suto, Ko Okumura, Haruhiko Taguchi, Kenji Matsumoto, Hirohisa Saito, Katsuko Sudo, Susumu Nakae

**Affiliations:** 1 Department of Immunology, Faculty of Health Sciences, Kyorin University, Tokyo, 181-8612 Japan; 2 Department of Allergy and Clinical Immunology, National Research Institute for Child Health and Development, Tokyo, 157-8535 Japan; 3 Graduate School of Integrated Sciences for Life, Hiroshima University, 1-4-4 Kagamiyama, Higashi-Hiroshima City, Hiroshima, 739-0046 Japan; 4 Department of Immune Regulation, Research Institute, National Center for Global Health and Medicine, Chiba, 272-8516 Japan; 5 Laboratory for Anatomy and Cell Biology, Department of Health Sciences, Kyorin University, Tokyo, 181-8612 Japan; 6 Laboratory of Systems Biology, Center for Experimental Medicine and Systems Biology, The Institute of Medical Science, The University of Tokyo, Tokyo, 108-8639 Japan; 7 Department of Analytical Chemistry, Faculty of Health Sciences, Kyorin University, Tokyo, 181-8612 Japan; 8 Atopy Research Center, Juntendo University School of Medicine, Tokyo, 113-8412 Japan; 9 Animal Research Center, Tokyo Medical University, Tokyo, 160-8402 Japan; 10 Precursory Research for Embryonic Science and Technology (PRESTO), Japan Science and Technology Agency, Saitama, 332-0012 Japan

**Keywords:** Interleukins, Acute inflammation, Allergy, Asthma

## Abstract

Exposure to various antigens derived from house dust mites (HDM) is considered to be a risk factor for development of certain allergic diseases such as atopic asthma, atopic dermatitis, rhinitis and conjunctivitis. Chitin is an insoluble polysaccharide (β-(1–4)-poly-*N*-acetyl-d-glucosamine) and a major component in the outer shell of HDMs. Mice exposed to chitin develop asthma-like airway eosinophilia. On the other hand, several lines of evidence show that the effects of chitin on immune responses are highly dependent on the size of chitin particles. In the present study, we show that chitin induced production of IL-33 and TSLP by alveolar and bronchial epithelial cells, respectively, in mice. IL-25, IL-33 and TSLP were reported to be important for group 2 innate lymphoid cell (ILC2)-, but not Th2 cell-, dependent airway eosinophilia in a certain model using chitin beads. Here, we show that—in our murine models—epithelial cell-derived IL-33 and TSLP, but not IL-25, were crucial for activation of resident lung Th2 cells as well as group 2 innate lymphoid cells (ILC2s) to produce IL-5, resulting in development of chitin-induced airway eosinophilia. Our findings provide further insight into the underlying mechanisms of development of HDM-mediated allergic disorders.

## Introduction

Allergic asthma is one of the most common chronic inflammatory diseases of the airways, characterized by paroxysmal wheezing, respiratory distress and cough. It causes significant morbidity and mortality, with almost 300 million cases worldwide, accounting for a significant portion of overall healthcare costs^1,2^. Various types of airborne allergens derived from plants, fungi, arthropods and animals are considered to be risk factors for allergic asthma^3^. Antigens derived from house dust mites (HDM) are major allergens for the development of atopic asthma as well as atopic dermatitis, rhinitis and conjunctivitis^4^. In particular, HDM-derived cysteine proteases such as Der p/f1 can disrupt adhesion molecules that form tight junctions between epithelial cells, allowing antigens to enter the body^5–7^. Moreover, Der p/f1′s protease activity also causes epithelial cells to produce inflammatory cytokines via protease-activated receptors^8^. In addition, protease allergens are known to induce necrosis of epithelial cells, leading to release cellular components, including damage-associated molecular patterns (DAMPs) such as HMGB-1, IL-1α, IL-33, uric acid and ATP, and induction of local inflammation via activation of innate immune systems^9,10^. Indeed, exposure to a plant-derived cysteine protease, papain, which is homologous to Der p/f1, leads to development of asthma-like airway inflammation in humans^11^. In mice, papain activates pulmonary epithelial cells to produce IL-33, which can induce type 2 cytokines. This results in development of type 2 cytokine-dependent airway eosinophilia through activation of group 2 innate lymphoid cells (ILC2s) and basophils, even in the absence of adaptive immune cells^12–15^.

In addition to protease allergens, exposure to chitin, which is an insoluble polysaccharide (β-(1–4)-poly-*N*-acetyl-d-glucosamine) and a major component in the outer shell of HDMs, also can lead to development of asthma-like airway eosinophilia through activation of innate immune cells, even in the absence of adaptive immune cells^16^. In the setting, chitin induces production of IL-25 and TSLP, in addition to IL-33, by airway epithelial cells, followed by activation of ILC2s to produce IL-5 and IL-13^17^. Chitin also enhances antigen-specific Th2 cell polarization by promoting cytokine production by lung dendritic cells (DCs) in response to IL-33, contributing to aggravation of Th2 cell-mediated allergic airway eosinophilia^18^. On the other hand, several lines of evidence show that the effects of chitin on immune responses are highly dependent on the size of chitin particles^19–21^. However, the size-dependent effects of chitin on airway inflammation in mice remain unclear, especially when it is administered intranasally. In the present study, we investigated the effects of different sizes of chitin particles on chitin-mediated airway eosinophilia in mice.

## Results

### Investigation of size-dependent effects of chitin on airway inflammation

**Figure 1:**
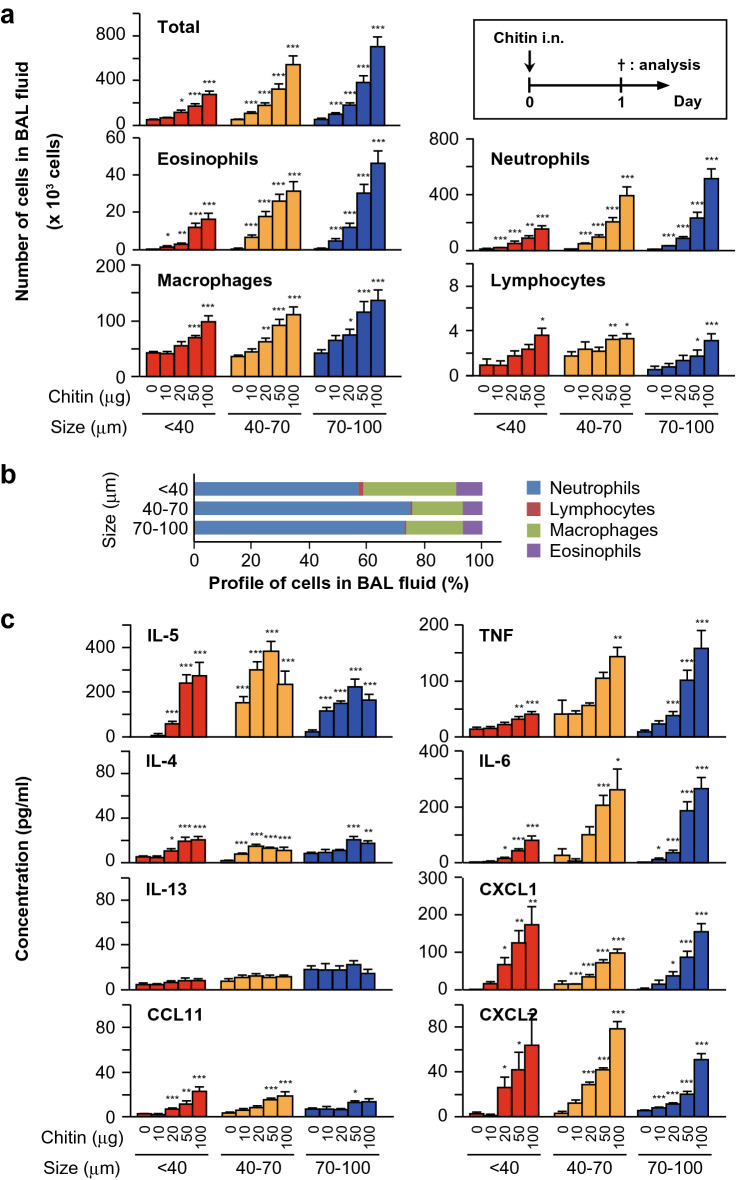
Investigation of size-dependent effects of chitin on airway inflammation. C57BL/6J-wild-type mice were intranasally treated one time with different doses (0, 10, 20, 50 and 100 μg per mice) of chitin (small: < 40 μm; medium: 40–70 μm; large: 70–100 μm) suspended in saline. Twenty-four hours after the inhalation of chitin or saline alone (= 0 μg of chitin), the BAL fluid was collected. (**a**) The total cells and each type of cell (eosinophils, neutrophils, macrophages and lymphocytes) in the BAL fluids were counted. (**b**) The proportions of leukocytes in the BAL fluids in (**a**) are shown. (**c**) The levels of cytokines and chemokines in the BAL fluids were measured by ELISA. Data from 2 independent experiments were pooled and shown as the mean + SE (n = 10). *P < 0.05, **P < 0.01, ***P < 0.005 *vs*. saline alone (= 0 μg of chitin).

Several lines of evidence show that the effects of chitin on immune responses are highly dependent on the size of chitin particles^19–21^. Nevertheless, the size-dependent effects of chitin on airway inflammation in mice remain unclear, especially when it is intranasally administered to mice. Our initial experiments were conducted in C57BL/6N-wild-type mice. The mice received a single intranasal treatment with different sizes (small: < 40 μm; medium: 40–70 μm; and large: 70–100 μm) and doses (0, 10, 20, 50 and 100 μg per mouse) of chitin particles suspended in saline. Twenty-four hours later, the total cells and each type of cell (eosinophils, neutrophils, macrophages and lymphocytes) in BAL fluids were counted and found to be dose-dependently increased with each chitin size (Fig. 1a). That is, the maximum response was observed in mice treated with the largest chitin dose (100 μg per mouse), irrespective of the chitin particle size (Fig. 1a). Neutrophils were the major cell population in the BAL fluids and were–3–tenfold greater than the number of eosinophils, regardless of the chitin size and dose (Fig. 1a). Although the leukocyte profiles in the BAL fluids were similar with 100 μg of chitin in all sizes (Fig. 1b), the numbers of neutrophils and eosinophils in BAL fluids from mice treated with the large size (70–100 μm) of chitin were significantly larger than those with the small size (< 40 μm) of chitin (p < 0.05). They also tended to be larger than with the medium size (40–70 μm) of chitin, although the difference was not statistically significant (Fig. 1a).

Type 2 cytokines (IL-4, IL-5, IL-13, etc.) contribute to eosinophilic inflammation, while type 3 cytokines (IL-17A, IL-17F, etc.) and IL-1, IL-6 and TNF contribute to neutrophilic inflammation. We next examined the levels of those cytokines as well as certain chemokines in BAL fluids from mice, as shown in Fig. 1c. The levels of IL-4 and IL-5, but not IL-13, in the BAL fluids were significantly increased in mice treated with 10–100 μg of each size of chitin compared with mice treated with saline (= 0 μg of chitin) (Fig. 1c). The BAL fluid levels of CCL11 (also called eotaxin), which is a chemoattractant for eosinophils, were significantly, but not drastically, increased in mice treated with 100 μg of each size of chitin compared with the saline control mice (Fig. 1c). IL-6, TNF and such neutrophil chemoattractants as CXCL1 (also called KC) and CXCL2 (also called MIP-2) in the BAL fluids were significantly and dose-dependently increased in mice treated with each size of chitin compared with saline (Fig. 1c). The increased levels of IL-6 and TNF in the BAL fluids from mice treated with all doses of the medium (40–70 μm) and large (70–100 μm) sizes of chitin were nearly equivalent, and greater than with the small size (< 40 μm) of chitin (Fig. 1c). On the other hand, the levels of CXCL1 and CXCL2 were nearly comparable regardless of the chitin size and dose (Fig. 1c). IL-17A, IL-17F and IL-1β were below the limit of detection by ELISA with all treatments (data not shown).

Based on these experimental results, 100 μg of chitin, which induced maximum airway eosinophilia and neutrophilia, was used in subsequent experiments.

**Figure 2:**
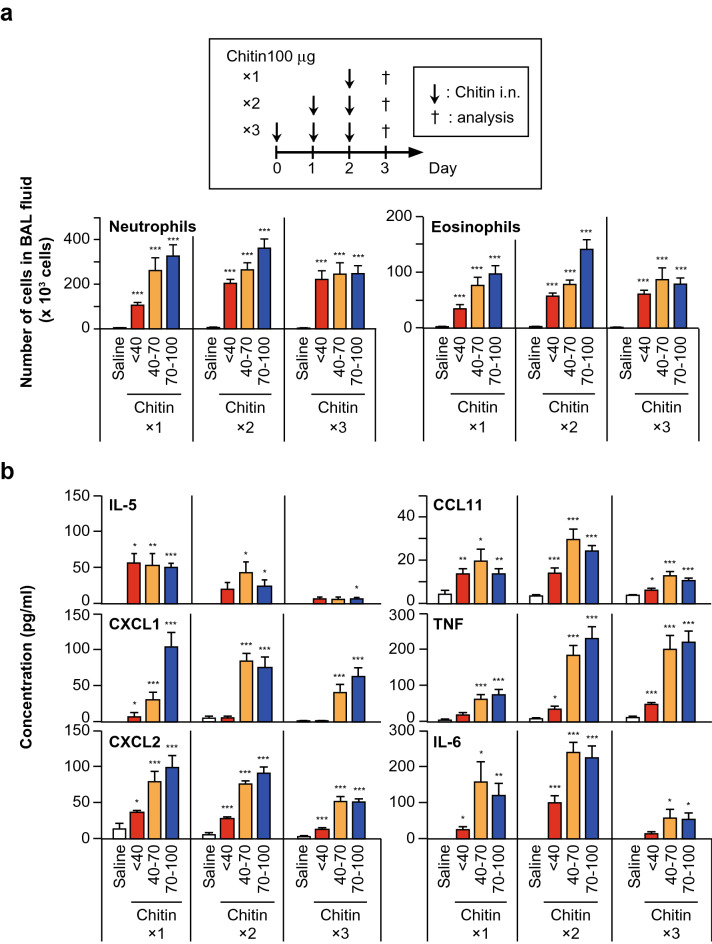
Induction of robust airway inflammation in mice by a single inhalation of chitin. C57BL/6 J-wild-type mice were intranasally treated one, two or three times with 100 μg of chitin (small: < 40 μm; medium: 40–70 μm; large: 70–100 μm) suspended in saline or with saline alone. Twenty-four hours after the last inhalation of chitin or saline alone, BAL fluids and lungs were collected. (**a**) Each type of cell (eosinophils, neutrophils) in the BAL fluids were counted. (**b**) The levels of cytokines and chemokines in the BAL fluids were measured by ELISA. Data from 2 independent experiments were pooled and shown as the mean + SE (n = 10). *P < 0.05, **P < 0.01, ***P < 0.005 *vs*. saline alone (= 0 μg of chitin).

**Figure 3:**
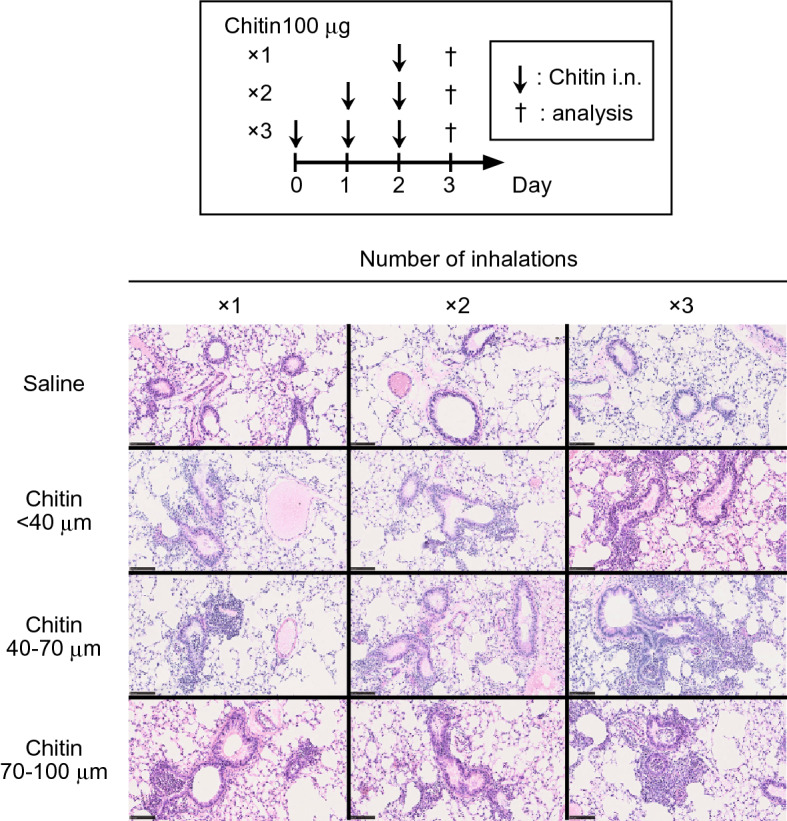
Histological changes in the lungs after inhalation of chitin. Lungs were harvested as in Fig. 2. Lung sections were stained with hematoxylin and eosin. Scale bar = 250 μm. Data show a representative result from the lungs of 5 mice.

We next examined the effect of the number of inhalations of each chitin particle size on induction of airway inflammation. C57BL/6N-wild-type mice were treated intranasally once, twice or three times with 100 μg of chitin particles of different size (small: < 40 μm; medium: 40–70 μm; and large: 70–100 μm) suspended in saline. Twenty-four hours after the last inhalation, the numbers of neutrophils and eosinophils, as well as the levels of cytokines (IL-5, IL-6 and TNF) and chemokines (CCL11, CXCL1 and CXCL2), in the BAL fluids from mice treated twice or three times with each size of chitin were not markedly increased compared with a single inhalation (Fig. 2a,b). In histological analysis by HE staining, although leukocytes had infiltrated around the bronchi with each size of chitin, the severity of lung inflammation did not differ dramatically, irrespective of the chitin size and number of inhalations (Fig. 3). Reflecting the low levels of IL-13 in the BAL fluids (Fig. 1b), PAS-positive cells were hardly detected in the lungs, regardless of the chitin size/dose and number of inhalations (data not shown). Taken together, these observations suggest that a single inhalation of chitin induced robust airway inflammation in mice as assessed by the numbers of inflammatory cells in the BAL fluid. Therefore, a single chitin inhalation was used in subsequent experiments.

### Importance of IL-5 for induction of airway eosinophilia by large-size chitin particles

**Figure 4:**
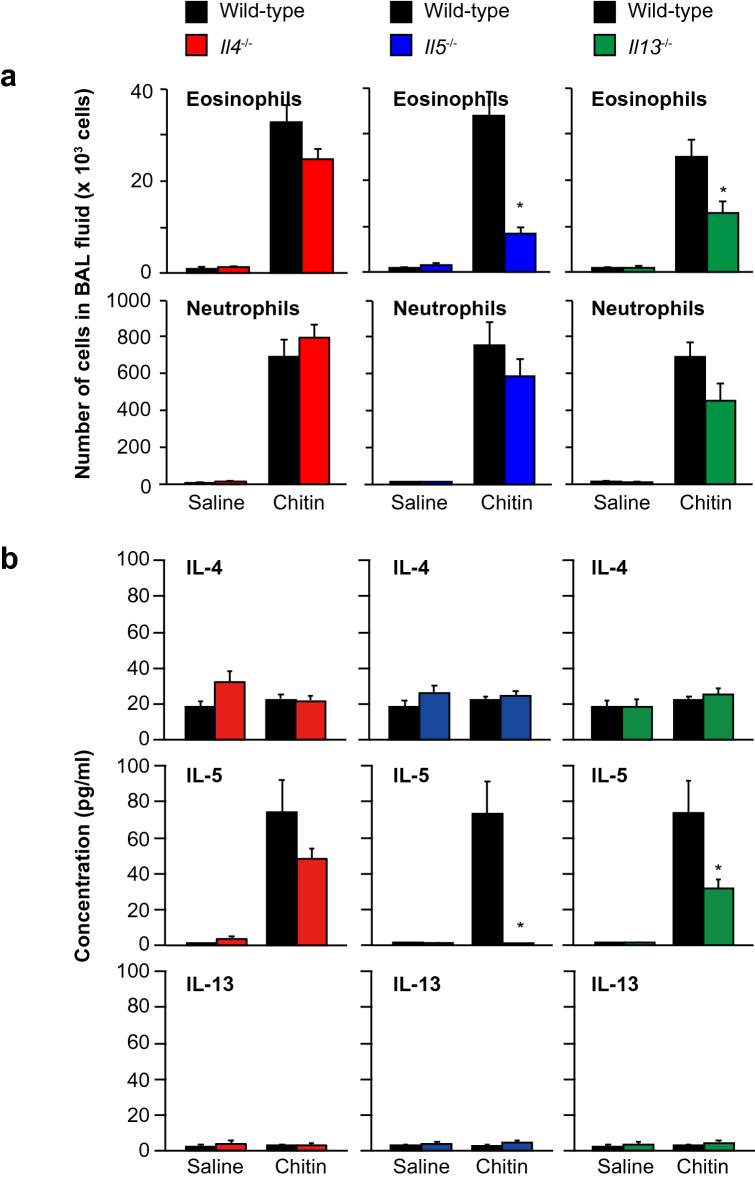
Importance of IL-5 for large-size chitin-induced airway eosinophilia. C57BL/6J-wild-type (n = 18–28), *Il4*^−/−^ (n = 27), *Il5*^−/−^ (n = 15) and *Il13*^−/−^ (n = 13) mice were intranasally treated one time with 100 μg of chitin (large size: 70–100 μm) suspended in saline or with saline alone. Twenty-four hours later, BAL fluids were collected. (**a**) The eosinophils and neutrophils in the BAL fluids were counted. (**b**) The levels of IL-4, IL-5 and IL-13 in the BAL fluids were measured by ELISA. Data from 2 independent experiments were pooled and shown as the mean + SE *P < 0.05 *vs*. wild-type mice.

Van Dyken, et al. demonstrated that two type 2 cytokines, i.e., IL-5 and IL-13, are important for induction of airway eosinophilia in mice treated intranasally with 50–70–μm-diameter chitin beads (5000 beads per mouse, once per day for 2 days)^17^. Thus, we examined the contribution of such type 2 cytokines as IL-4, IL-5 and IL-13 to induction of airway eosinophilia in mice using the large-size chitin particles (70–100 μm). C57BL/6-wild-type, -*Il4*^−/−^, -*Il5*^−/−^ and -*Il13*^−/−^ mice were treated intranasally once with 100 μg of the large-size chitin (70–100 μm). Twenty-four hours later, the number of eosinophils in the BAL fluid was markedly decreased in *Il5*^−/−^ mice, and partially, but significantly, reduced in *Il13*^−/−^ mice compared with the wild-type mice (Fig. 4a). However, eosinophils in the BAL fluid from *Il4*^−/−^ mice were comparable to in the wild-type mice (Fig. 4a). In association with this, the level of IL-5 in the BAL fluid was reduced in *Il13*^−/−^ mice—but normal in *Il4*^−/−^ mice—compared with the wild-type mice (Fig. 4b). Consistent with Fig. 1c, IL-4 and IL-13 were not elevated in the BAL fluid in any group (Fig. 4b). In contrast to eosinophils, the numbers of neutrophils in the BAL fluids were comparable in these mice (Fig. 4a). These results indicate that IL-5 is potently involved in induction of airway eosinophilia, but not neutrophilia, in mice treated intranasally with large-size chitin particles (70–100 μm).

### Importance of ILC2s for induction of airway eosinophilia by large-size chitin particles

**Figure 5:**
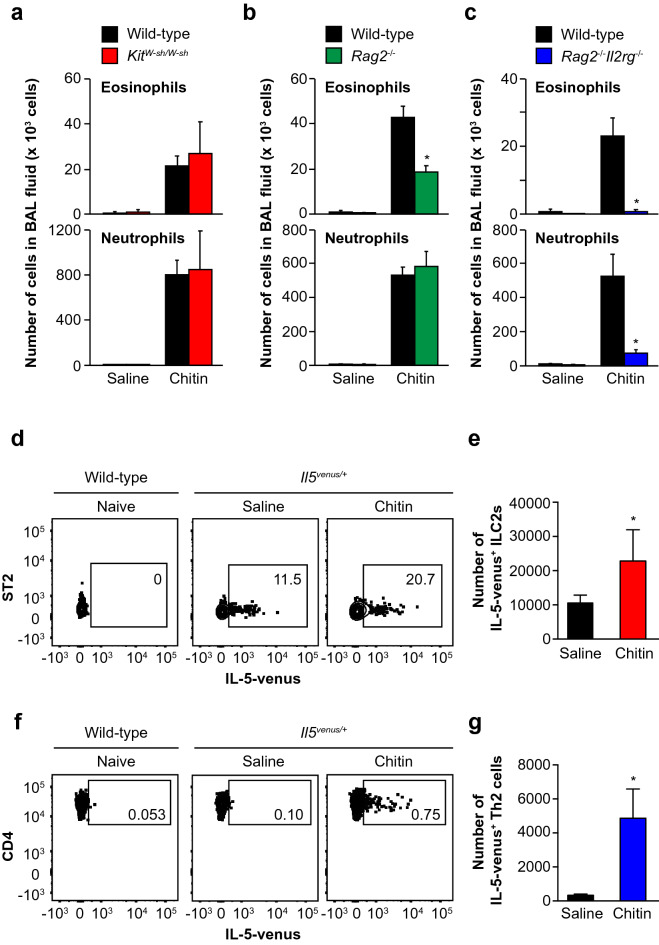
Importance of ILC2s for large-size chitin-induced airway eosinophilia. Mice were intranasally treated one time with 100 μg of chitin (large size: 70–100 μm) suspended in saline or with saline alone. Twenty-four hours later, BAL fluids and lungs were collected. (**a**) Numbers of eosinophils and neutrophils in the BAL fluids from C57BL/6N-wild-type (n = 9) and *Kit*^*W-sh/W-sh*^ (n = 4) mice. (**b**) Numbers of eosinophils and neutrophils in the BAL fluids from C57BL/6J-wild-type (n = 22) and *Rag2*^−/−^ (n = 19) mice. (**c**) Numbers of eosinophils and neutrophils in the BAL fluids from C57BL/6J-wild-type (n = 10) and *Rag2*^−/−^*Il2rg*^−/−^ (n = 8) mice. (**d**) Proportion of IL-5-venus-positive cells in CD45^+^ CD25^+^ lineage marker-negative ST2^+^ cells (ILC2s) in the lungs from naïve wild-type and saline- and chitin-treated *Il5*^*venus/*+^ mice by FACS. (**e**) Number of ILC2s in the lungs from saline- and chitin-treated *Il5*^*venus/*+^ mice (n = 5). (**f**) Proportion of IL-5-venus-positive cells in CD45^+^ CD3^+^ CD4^+^ T cells in the lungs from naïve wild-type and saline- and chitin-treated *Il5*^*venus/*+^ mice by FACS. (**g**) Number of Th2 cells in the lungs from saline- and chitin-treated *Il5*^*venus/*+^ mice (n = 5). (**a**–**c**,**e**,**g**) Data from 2 independent experiments were pooled and shown as the mean + SE *P < 0.05 *vs*. wild-type mice. (**d**,**f**) Data show a representative result from the lungs of 5 mice. (**d**,**f**) Cells were analyzed on a FACS Aria II Cell Sorter (BD Biosciences) with BD FACSDiva v6.1.3 software (BD Biosciences) and FlowJo v10.5.3 software (BD Biosciences; https://www.flowjo.com/solutions/flowjo).

IL-5 and/or IL-13 are produced by various types of immune cells including Th2 cells, NKT cells, ILC2 cells and/or mast cells. Mast cells’ contribution to induction of chitin-mediated eosinophilia has been suggested to be dependent on the size of chitin particles^16,21^. In our experiment (one inhalation of large-size chitin particles (70–100 μm)), the numbers of eosinophils as well as neutrophils in the BAL fluid were comparable between mast-cell-deficient *Kit*^*W-sh/W-sh*^ mice and the wild-type mice (Fig. 5a). These results indicate that mast cells (and mast-cell-derived type 2 cytokines) are not essential for induction of airway eosinophilia in the setting.

The BAL fluid of *Rag2*^−/−^ mice, which lack T cells, B cells and NKT cells, contained a partially reduced number of eosinophils, but a normal number of neutrophils, compared with the wild-type mice 24 h after the inhalation of large-size chitin (70–100 μm) (Fig. 5b). On the other hand, the numbers of both eosinophils and neutrophils in the BAL fluid were greatly reduced in *Rag2*^−/−^ *Il2rg*^−/−^ mice, which lack ILCs as well as *rag*-dependent acquired immune cells, compared with the wild-type mice 24 h after the inhalation (Fig. 5c). These observations suggest that ILCs are the immune cells most responsible for induction of both eosinophilia and neutrophilia in the BAL fluid of mice 24 h after the inhalation of large-size chitin (70–100 μm).

We next identified the IL-5- and IL-13-producing cells in the lungs of IL-5-reporter (venus) and IL-13-reporter (td-tomato) mice 24 h after inhalation of large-size chitin (70–100 μm). Expression of IL-5 (as venus-positive) and IL-13 (as td-tomato-positive) was hardly detectable in CD45-negative non-immune cells of the lungs from saline- and chitin-treated mice (data not shown). On the other hand, expression of IL-5 was increased in CD45^+^ CD90^+^ lin^−^ ST2^+^ ILC2s (Fig. 5d) and CD45^+^ CD90^+^ CD3^+^ CD4^+^ Th2 cells (Fig. 5f) in the total lung cells from chitin-treated IL-5-reporter mice compared with saline-treated IL-5-reporter mice and naive wild-type mice (non-reporter mice as negative controls). The numbers of IL-5-producing ILC2s (Fig. 5e) and Th2 cells (Fig. 5g) were significantly increased in the total lung cells from chitin-treated IL-5-reporter mice compared with saline-treated IL-5-reporter mice. However, expression of IL-13 was hardly detected in CD45-negative non-immune cells, or CD45^+^ CD90^+^ lin^−^ ILCs and CD45^+^ CD90^+^ lin^+^ immune cells, in the total lung cells from IL-13-reporter mice, irrespective of chitin treatment (data not shown). These observations suggest that ILC2s and Th2 cells are major producers of IL-5, contributing to induction of airway eosinophilia in mice after inhalation of large-size chitin (70–100 μm).

### Importance of TSLP and IL-33, but not IL-25, for induction of airway eosinophilia by large-size chitin particles

**Figure 6:**
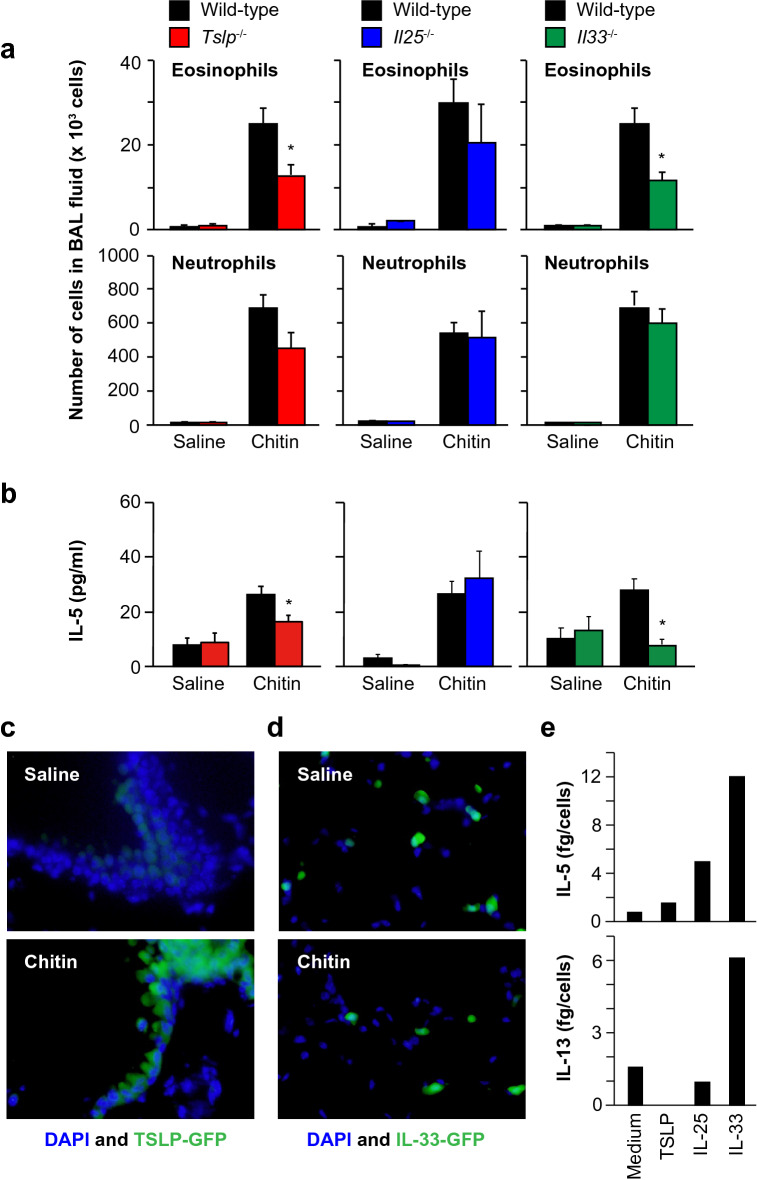
Importance of TSLP and IL-33, but not IL-25, for large-size chitin-induced eosinophilia. Mice were intranasally treated one time with 100 μg of chitin (large size: 70–100 μm) suspended in saline or with saline alone. Twenty-four hours later, BAL fluids and lungs were collected. (**a**) Numbers of eosinophils and neutrophils in the BAL fluids from C57BL/6N-wild-type (n = 16–26), *Tslp*^−/−^ (n = 22), *Il25*^−/−^ (n = 17) and *Il33*^−/−^ (n = 17) mice. (**b**) IL-5 levels in the BAL fluids of mice in (**a**). (**c**) Detection of TSLP expression in bronchial epithelial cells by IHC. (**d**) Detection of IL-33 expression in alveolar epithelial cells by IHC. (**e**) IL-5 and IL-13 levels in the culture supernatants of ILC2s in the presence and absence of TSLP, IL-25 and IL-33. (**a**,**b**) Data from 2 independent experiments were pooled and shown as the mean + SE *P < 0.05 *vs*. wild-type mice. (**c**,**d**) Data show a representative result from the lungs of 3 mice. (**e**) Data show a representative result from 2 independent experiments, which gave similar results.

TSLP, IL-25 and/or IL-33 are key stimulators for ILC2s to induce IL-5, contributing to induction of eosinophilia^22,23^. Therefore, we examined whether TSLP, IL-25 and/or IL-33 contribute to induction of eosinophilia in the BAL fluid of mice after inhalation of large-size chitin (70–100 μm). Eosinophils were significantly reduced in the BAL fluid of *Tslp*^−/−^ and *Il33*^−/−^ mice compared with the wild-type mice, whereas their numbers were comparable between *Il25*^−/−^ and wild-type mice (Fig. 6a). In association with this, the IL-5 level was significantly decreased in the BAL fluid of those *Tslp*^−/−^ and *Il33*^−/−^ mice, but normal in *Il25*^−/−^ mice, compared with the wild-type mice (Fig. 6b). On the other hand, the numbers of neutrophils in the BAL fluid were comparable in all groups (Fig. 6a). These results indicate that TSLP and IL-33, but not IL-25, are crucial for induction of airway eosinophilia in mice after inhalation of large-size chitin particles (70–100 μm).

We next identified the TSLP- and IL-33-producing cells in the lungs of *Tslp*^−/−^ and *Il33*^−/−^ mice, which express EGFP instead of TSLP and IL-33, respectively, after inhalation of chitin or saline. TSLP expression was preferentially increased in bronchial, but not alveolar, epithelial cells of the lungs of chitin-treated mice compared with saline-treated mice (Fig. 6c). On the other hand, IL-33 was constitutively expressed in alveolar, but not bronchial, epithelial cells, irrespective of chitin or saline treatment (Fig. 6d). Thus, these findings suggest that TSLP and IL-33, which are produced by different epithelial cells of the lungs, may contribute to lung ILC2 activation after induction of airway eosinophilia in mice by inhalation of large-size chitin particles (70–100 μm). To elucidate this, we sorted ILC2s from the lungs of chitin-treated mice, and then, cultured the cells in the presence and absence of recombinant TSLP, IL-25 and IL-33. IL-33, but not TSLP or IL-25, strongly induced IL-5 and IL-13 production by the sorted ILC2s in vitro (Fig. 6e). On the other hand, TSLP may be important for type 2 cytokine production by resident Th2 cells rather than by ILC2s.

## Discussion

Several lines of evidence show that chitin’s effects on immune responses are highly dependent on its size^19–21^. For example, iNOS and cox-2 expression was increased in macrophage-like cells (RAW264.7 cells) in response to a phagocytizable size of chitin (1–10 μm), but not non- phagocytizable chitin (> 50 μm)^21^. Conversely, IL-10 and/or TNF production increased in thioglycolate-induced mouse peritoneal macrophages in response to non- phagocytizable chitin (< 40 μm and/or 70–100 μm), but not phagocytizable chitin (< 2 μm)^19^. Moreover, IL-17 production was induced in thioglycolate-induced mouse peritoneal macrophages in response to 40–70–μm chitin, but not 70–100–μm chitin^24^.

In in vivo analysis, inhalation of chitin alone induced airway inflammation in mice. In addition to the size of chitin, it was suggested that the number of administrations of chitin and the analysis points may have different effects on the immune responses^24^. For example, Da Silva, et al. reported that a single inhalation of chitin (40–70 μm; 25 μg) by mice resulted in airway neutrophilia 6 h later^24^. On the other hand, Reese, et al. reported that two inhalations of chitin (unknown size and dose) by mice resulted in airway eosinophilia 24 h after the last inhalation^16^. Van Dyken, et al. reported that two inhalations of chitin (50–70 μm; 5000 beads) by mice resulted in airway neutrophilia or eosinophilia 4 or 24 h after the last inhalation^17^. Therefore, in the present study, we first performed optimization of the protocol for achieving chitin-induced airway inflammation, as shown in Figs. 1 and 2. We tested various doses of chitin (10, 20, 50 and 100 μg per mouse) and found that 100 μg was the most effective dose in inducing airway inflammation, irrespective of the chitin size (Fig. 1a). Regarding the number of inhalations of chitin, one inhalation resulted predominantly in neutrophilia, while two and three inhalations of chitin resulted in both neutrophilia and eosinophilia, irrespective of the chitin size (Fig. 2a). Large-size chitin (70–100 μm) was the most effective in inducing airway inflammation (Figs. 1a, 2a).

Van Dyken, et al. reported that IL-33, IL-25 and TSLP were crucial for activation of ILC2s, but not Th2 cells, to induce IL-5 and IL-13 following development of airway eosinophilia in mice 24 h after chitin inhalation (50–70 μm; 5000 beads, obtained from New England BioLabs, Inc.)^17^. In our present study, IL-33 and TSLP, but not IL-25, were crucial for activation of not only ILC2s but also Th2 cells to induce IL-5 and IL-13 following development of airway eosinophilia in mice 24 h after chitin inhalation (70–100 μm; 100 μg) (Figs. 4–6). As far as we tested, the dry weight of 5000 chitin beads (New England BioLabs, Inc.) was approximately 875 μg. Mice treated intranasally with 100 μg of chitin beads (New England BioLabs, Inc.) did not develop airway inflammation (data not shown). The chitin beads from New England BioLabs were spherical^21^, whereas our chitin particles (70–100-μm) had various shapes (data not shown). In addition to the size of chitin, its shape, dose and number of inhalations may have different effects on the immune responses.

In various models of airway inflammation in mice, IL-33, IL-25 and/or TSLP showed increased expression in the lungs after inhalation of antigens. Indeed, after chitin inhalation the levels of those cytokines were increased in the lungs^2,17^. However, the exact cells producing those cytokines remained unclear. In the present study, we show for the first time that IL-33 was produced by alveolar epithelial cells, while TSLP was produced by bronchial epithelial cells, in the lungs after chitin inhalation (Fig. 6c). Chitin is a polysaccharide, not a protein antigen, suggesting that chitin-induced airway inflammation is independent of TCR-MHC class II interaction on T cells and DCs. Nevertheless, in our models, IL-5-producing Th2 cells and ILC2s were crucial for development of chitin-induced airway eosinophilia. A robust profile of resident CD4^+^ T cells was observed even in lungs from naïve wild-type mice (Fig. 5e). These observations suggest that TSLP and IL-33, which are produced by different epithelial cells of the lungs, activate Th2 cells and/or ILC2s to produce IL-5 independently of TCR-MHC class II interaction on T cells and DCs.

In summary, large-size chitin particles (70–100 μm) were the most effective in inducing airway inflammation. In addition to the size of chitin, its shape, dose and number of inhalations may have different effects on the immune responses. Alveolar epithelial-cell-derived IL-33 and bronchial epithelial-cell-derived TSLP are crucial for activation of resident ILC2s and/or Th2 cells in the lungs, which contributes to induction of IL-5 and IL-13-dependent airway eosinophilia after inhalation of large-size chitin (70–100 μm).

## Methods

### Mice

Wild-type mice (C57BL/6 J and C57BL/6 N; 6 to 12 weeks of age) were purchased from Japan SLC, Inc. (Shizuoka, Japan). C57BL/6 J-*Il4*^−/−^ mice and -*Il5*^−/−^ mice were obtained from Jackson Laboratory (Bar Harbor, ME, USA). C57BL/6*-Rag2*^−/−^ and *-Rag2*^−/−^*Il2rg*^−/−^ mice, C57BL/6*-Il13*^−/−^ and -*Il13*^*tomato/tomato*^ mice^25^, and C57BL/6-*Il5*^*venus/venus*^ mice^26^ were kindly provided by Drs. Hiromitsu Nakauchi (The University of Tokyo, Tokyo, Japan), Andrew N.J. McKenzie (MRC, UK), and Kiyoshi Takatsu (University of Toyama, Toyama, Japan), respectively. C57BL6N-*Il25*^−/−^ mice, C57BL/6 N-*Il33*^−/−^ mice and C57BL/6N-*Tslp*^−/−^ mice were generated as described elsewhere^12,27,28^. All mice were housed under a specific-pathogen-free environment at the Institute of Medical Science, The University of Tokyo, National Research Institute for Child Health and Development, and the Faculty of Health Sciences, Kyorin University. All animal experiments were approved and performed in accordance with the animal care and use committees of the institutes and universities (I16-01–04, Kyorin University; A2012-004-C05, the National Research Institute for Child Health and Development and A11-28 and A14-10, the Institute of Medical Science, The University of Tokyo).

### Chitin-induced airway inflammation

Chitin particles, i.e., < 40 μm, 40–70 μm, and 70–100 μm, were prepared as described previously^18^. In brief, chitin was partially hydrolyzed with 6 M hydrochloric acid for 30 min at 60 °C, followed by neutralization with 1 M Tris–HCl buffer (pH 8.0) and centrifugation at 3000 rpm for 5 min at room temperature. After washing with deionized and sterilized water, the chitin was routinely passed through a 100-μm nylon mesh filter and fractionated using size-exclusion nylon mesh filters into 70–100 μm, 40–70 μm and < 40 μm particles. The chitin particles were freeze-dried and resuspended in water at the appropriate concentration, sterilized by autoclaving and stored at 4 °C.

Mice were intranasally treated with 10–100 μg chitin in saline or with saline alone, one to three times. One day after the last treatment, the lungs and bronchoalveolar lavage (BAL) fluid were harvested.

### Bronchoalveolar lavage fluids

Twenty-four hours after the last challenge with chitin or saline, BAL fluids were collected as described elsewhere^18^. The BAL fluids were centrifuged, and the BAL cells were resuspended in 200 μl of HBSS supplemented with 2% FCS. The number of each cell type among the BAL cells was counted with an automated hematology analyzer (XT-1800i; Sysmex, Hyogo, Japan), according to the manufacturer’s instructions.

### ELISA for cytokines

The levels of cytokines and chemokines in the BAL fluids were determined using ELISA kits for mouse IL-4, IL-5, IL-6, IL-13, TNF-α, KC/CXCL1, MIP-2/CXCL2 and eotaxin/CCL11 (eBioscience, Biolegend or PeproTech). All procedures were performed according to the manufacturers’ instructions.

### Histology

Twenty-four hours after the last chitin or saline inhalation, lungs were harvested and fixed in Carnoy’s solution. The fixed tissues were embedded in paraffin, sliced into 3-µm sections, and subjected to hematoxylin and eosin staining or periodic acid-Schiff staining.

### Immunohistochemistry

Detection of IL-33 and TSLP by immunohistochemistry was performed as described elsewhere with minor modifications^29^. Twenty-four hours after the last chitin inhalation, the trachea was cannulated with a 22-G blunt needle attached to a syringe, and 4% paraformaldehyde in 0.05 M phosphate buffer (pH 7.4) was infused. The lungs were immediately harvested and immersed in a fixative solution consisting of 4% paraformaldehyde in 0.05 M phosphate buffer (pH 7.4) at 4 °C for 20–24 h. The tissues were then immersed in 30% sucrose in 0.05 M phosphate buffer (pH 7.4) at 4 °C for more than 2 days, embedded in Tissue Tek OCT compound (Sakura Finetek Japan, Tokyo, Japan), and rapidly frozen. Frozen frontal and horizontal sections (8-μm thickness) were prepared using a cryostat (Tissue-tek Polar DM; Sakura Finetek Japan) and mounted on slide glasses (Matsunami, Osaka, Japan). Nuclei were counterstained by incubation with Vectashield Mounting Medium containing 4,6-diamidino-2-phenylindole (DAPI; Vector Laboratories, Burlingame, CA, USA). Sections were scanned using a fluorescence microscope (cellSens Dimension System; Olympus, Tokyo, Japan). Each section was scanned at least three times.

### Flow cytometry

The lung cells were prepared and analyzed as described elsewhere with minor modifications^18^. The lungs were minced and digested in HBSS containing 1% BSA, 200 U/ml collagenase type 5 (Worthington Biochemical Corporation), 1500 U/ml hyaluronidase (Worthington Biochemical Corporation) and 100 U/ml DNase I (Worthington Biochemical Corporation) for 30 min at 37 °C. After the digested lungs were homogenized with gentleMACS Dissociator (Miltenyi Biotec), the dissociated lung cells were collected by passing through a 70-μm nylon mesh. For ILC2s, the dissociated lung cells were incubated (20 min on ice) with anti-mouse CD16/CD32 mAb (93; BioLegend) in FACS buffer (HBSS containing 2% FCS) for FcR blocking. The cells were then incubated (30 min on ice) with a mixture of Ab cocktails containing APC-conjugated anti-mouse ST2 mAb (DJ8; MD Biosciences), APC/Cy7-conjugated anti-mouse CD45 mAb (30-F11; BioLegend), PE/Cy7-conjugated anti-mouse CD25 mAb (3C7; BioLegend), and biotin-conjugated anti-mouse lineage marker mAbs (CD3ε [145-2C11; BioLegend], CD4 [GK1.5; BioLegend], CD8 [53–6.7; BioLegend], FcεRIα [MAR-1; BioLegend], CD11c [N418; BioLegend], CD19 [6D5; BioLegend], Ter119 [TER-119; BioLegend], and Gr-1 [RB6-8C5; BioLegend]) in the presence of 7-Amino-Actinomycin D (7-AAD). After washing, the cells were incubated (30 min on ice) with PE -conjugated streptavidin. For Th2 cells, the dissociated lung cells were incubated (30 min on ice) with a mixture of Ab cocktails containing APC/Cy7-conjugated anti-mouse CD45 mAb, PE-conjugated anti-mouse CD4 mAb, and APC-conjugated anti-mouse CD3ε mAb, in the presence of 7-AAD. After washing, the proportions of IL-5-venus^+^ cells among 7-AAD-negative CD45^+^ lineage^−^ CD25^+^ ST2^+^ cells (as activated ILC2s) and CD45^+^ CD3ε^+^ CD4^+^ cells (as Th2 cells) were analyzed on a FACS Aria II Cell Sorter (BD Biosciences) with BD FACSDiva v6.1.3 software (BD Biosciences) and FlowJo v10.5.3 software (BD Biosciences; https://www.flowjo.com/solutions/flowjo).

### ILC2 culture

Mice were intranasally treated with 100 μg of chitin (70–100 μm), and the lungs were collected after 24 h. Dissociated lung cells were prepared as described above and suspended in 30 ml of 30% Percoll in FACS buffer. Then the cell suspension was layered on 5 ml of 70% Percoll in FACS buffer. After centrifugation, the cells in the middle layer were collected and incubated (15 min on ice) with anti-mouse CD16/CD32 mAb in FACS buffer for FcR blocking. The cells were then incubated (30 min on ice) with biotin-conjugated anti-mouse lineage marker mAbs (CD3ε, CD4, CD8, CD11c, CD19, FcεRIα, Gr-1, and Ter119) as described above. After washing, the cells were resuspended in Streptavidin MicroBeads (Miltenyi Biotec) and incubated for 30 min on ice. After washing, the cells that were positive for the lineage markers were magnetically depleted using an AutoMACS Cell Separator (Miltenyi Biotec). The cells in the negative fraction were collected and then incubated (30 min on ice) with a mixture containing PerCP/Cy5.5-conjugated anti-mouse KLRG1(Killer cell Lectin-like Receptor G1) mAb (2F1/KLRG1; BioLegend), PE/Cy7-conjugated anti-mouse CD127 mAb (A7R34; BioLegend), APC-conjugated anti-mouse Sca-1 mAb (D7; BioLegend), BD Horizon BV510-conjugated anti-mouse CD45 mAb (30-F11; BioLegend), BV421-conjugated Streptavidin (BioLegend) and eBioscience Fixable Viability Dye eFluor 780 (Thermo Fisher Scientific). After washing, Viability Dye-negative CD45^+^ lineage^−^ Sca-1^+^ KLRG1^+^ CD127^+^ cells, i.e., ILC2s, were sorted using a FACS Aria II Cell Sorter (BD Biosciences) with BD FACSDiva v6.1.3 software (BD Biosciences). The ILC2s were resuspended in 200 μl of RPMI1640 medium supplemented with 10% FBS, 100 U/ml penicillin and 100 μg/ml streptomycin, and then cultured in the presence and absence of 50 ng/ml of each of recombinant mouse IL-25 (R&D Systems), recombinant mouse IL-33 (R&D Systems) and recombinant mouse TSLP (R&D Systems) in 96-well round-bottomed plates (680–1000 cells/well) at 37 °C in a 5.0% CO_2_ incubator for 2 days. Then the culture supernatants were collected for ELISA.

### Statistics

The statistical evaluation of the results was determined by the unpaired Student’s t test, two-tailed, using Excel (Microsoft, Redmond, WA). All results are shown as the mean ± SE. P values of < 0.05 were considered statistically significant.

## Data Availability

All data generated or analyzed during this study are included in this published article.
